# Self-Urinary Catheter Removal for Urology Day Cases: A Safe, Efficient, Sustainable, and Patient-Centred Approach

**DOI:** 10.7759/cureus.94256

**Published:** 2025-10-09

**Authors:** Enamur Rahman, Branimir Penev, Irtiza Bhatti, Kirsty Ellis, Muhammad Usman Javed, Annie Murphy, Alastair Henderson, Mark Cynk

**Affiliations:** 1 Urology, Maidstone and Tunbridge Wells NHS Trust, Maidstone, GBR

**Keywords:** healthcare cost savings, length of hospital stay, nhs carbon footprint, patient-reported outcomes, same-day discharge, self-removal of catheter, self-trial without catheter, sustainability in healthcare, urology day surgery, virtual ward monitoring

## Abstract

Introduction

Urology day procedures need a significant proportion of patients to have an indwelling catheter for a substantial time post-procedure. Self-catheter removal offers better patient care for both patients and healthcare, while contributing to the sustainability goals of the National Health Service (NHS).

Patient and methods

This project has been used to remove a urethral catheter at home for patients who need a catheter after their surgery for over 12 hours and are expected to pass urine naturally post-catheter removal. From June 2024 to July 2025, 180 patients (average age: 63.1; range: 35-85) were included to carry out this retrospective audit, excluding those with post-procedural complications needing clinical supervision, frail patients, patients living alone, and patients unable to use the mobile application.

Results

In the study cohort, all patients were successful in removing their catheter at home and did not need to attend the accident and emergency (A&E) within 30 days for catheter-related issues. In the initial 44 patients, we analysed patient feedback, and 96% of the patients were happy to refer others for the service, with no reported difficulty related to catheter removal. This project improved same-day discharge rates from 41% (91 out of 220 patients) to 55% (150 out of 210 patients) for the Holmium Laser Enucleation of Prostate (HoLEP) procedure, saving about 1.7 tons of carbon emissions in total, while potentially saving 20.32£ of travel cost for hospital attendance per patient.

Conclusion

Although self-removal of a catheter is endorsed by standard practice in England, this study shows the appropriateness of the service for urology day cases, as there is a knowledge gap in the existing published reports. Also, suggest potential implications in other indications of catheter removal.

## Introduction

With the challenge to become more efficient with healthcare resources, like the rest of the world, the National Health Service (NHS) in the United Kingdom (UK) is pursuing a shift towards increasing day cases to manage the ever-increasing burden of elective surgical care, where patients are discharged the same day after an elective procedure without an overnight hospital stay [1]. This effort has led to an increase in the number of day cases [2]. In 2013-14, this rate was only 13%; it then increased to 23% before the COVID-19 pandemic. Following a decline in the number of elective cases caused by the pandemic, the rates reached an all-time high of 31%, when the number of elective cases in England was the same as before the pandemic [3]. Moreover, day cases are environmentally friendly as they result in a significant reduction in healthcare-related carbon footprint [3]. Furthermore, it improves patient experience by enabling early recovery in a familiar environment [4].

Unlike the other specialities, the urology day procedures require patients to pass urine before being safely discharged. If unable, the patient might need clinical attention after a urinary catheter insertion [5]. Additionally, after a significant proportion of these procedures, patients may require a catheter to avoid the risk of urinary retention due to various reasons, such as clot retention or a post-surgical inflammatory response. Hence, the catheter removal service is indispensable to urology day surgeries. The primary option for this service is a hospital-based trial without a catheter (TWOC), which requires intensive support by the hospital and healthcare providers. However, it has been reported to be very resource-intensive. Also, it can be cumbersome for the patients. They need to travel to the hospital, park, and spend substantial time to avail the service. Conversely, it is often argued that the hospital resources can be better allocated to other patients if the TWOC service can be shifted to a safe non-hospital setup [6].

As a result, the proposal of the community TWOC was first reported in August 2018. After a pilot study in 2020-21, the idea was put into practice. It has been reported that TWOC conducted in community settings is effective, evidence-based, and can reduce pressures on hospitals [6]. But it can be time-intensive for the healthcare professionals. The bladder and bowel nursing services (continence services) in many parts of the UK are already under pressure due to the rising level of healthcare service demand [5].

As a result, an emerging approach of self-catheter removal has gained attention in recent years. It has been reported in the United States of America (USA) that, for urogynaecology procedures, when patients travel more than five miles to the hospital for catheter removal, it is cheaper for them to remove the catheter at home than an in-hospital voiding trial [7]. Evidence suggests that this method, following urogynaecology procedures and robotic-assisted laparoscopic prostatectomy (RARP), is not only feasible but also safe for patients, contributing positively to their recovery experience. Additionally, it presents a cost-effective solution for healthcare facilities by reducing the length of hospital stays and minimizing the need for in-person follow-ups related to catheter management. As healthcare continues to evolve, this practice offers a promising alternative that enhances patient autonomy and comfort while streamlining hospital resources [8]

Necessity of this study

Although there has been only one published article on the clinical implications of self-TWOC for RARP for urology patients, where patients can not be discharged the same day, the same-day urological procedures could use this service for a wider cohort of patients. While looking into the statistics, there have been 8,760 cases of prostatectomies in the year 2023, with an increase of 17% from 2022 in England [9]. In comparison, there has been no such recent consolidated data for the number of urology day-case numbers, as it comprises a wide mixture of procedures. But just for the sake of comparison of the scale, there have been roughly 20,000 bladder outflow obstruction (BOO) cases in England every year on average from 2017 to 2022 [2], which invariably need a catheter after the procedure. Another procedure accounting for a substantial portion of the urology day-case loads is transurethral resection of bladder tumour (TURBT) surgeries. The most recent figures showed an average of nearly 20,000 TURBTs per year from 2013 to 2022 in England [3], a significant portion of whom need post-procedure catheterization. Again, day-case surgery reduces hospital bed use, lowers healthcare costs, and improves efficiency and patient throughput. It also enhances the patient experience through shorter hospital stays and quicker recovery, while contributing to sustainability by lowering the carbon footprint of care.

Hence, there has been a need for a study to assess self-TWOC for urology day cases. In this report, the authors try to evaluate the feasibility, safety, patient experience, and sustainability of self-catheter removal for patients undergoing urology day procedures.

## Materials and methods

Ethical considerations

Following planning and approval, the self-TWOC programme was introduced in July 2024 at Maidstone and Tunbridge Wells NHS Trust for the urology day cases. It was formally approved by the Trust following the recommendation of the Getting It Right First Time (GIRFT), an NHS England improvement programme. This study was registered in the clinical audit department locally.

Patient selection and data extraction

This was a retrospective analysis of the patients who had undergone self-TWOC at Maidstone and Tunbridge Wells NHS Trust. After their day cases, patients were enrolled for self-TWOC, where the patient would require more than 12 hours of catheterization following their elective surgery. They were only included if they were expected to urinate without the need for catheterization following the procedure by the operating surgeon. Patients with a previous history of retention were excluded from the study. Similarly, those who are living alone or very frail or who were not eligible to TWOC due to post-operative emergencies (e.g., clot retention, changing decision, had catheter removal before the self-TWOC attempt by either hospital or community nursing team, as was convenient) were excluded from the programme. All patients who underwent self-TWOC in the study period were selected from the data provided by the short-stay surgery database folder kept for self-TWOC patients. The patient demographic data were collected from the electronic health record (EHR). The primary outcome to determine a successful TWOC was a successful removal of the catheter without acute retention of urine. The secondary outcome was determined to assess accident and emergency (A&E) attendance for catheter-related issues, such as urinary tract infection (UTI), haematuria, and retention in <30 days. 

Provisions for the patients

Following same-day discharge with a catheter, the virtual ward service was used to monitor these patients. They were enlisted in an artificial intelligence-based healthcare platform used by the virtual ward. As a result, a patient/their carer who cannot use a smartphone-based application for this purpose could not be included in our study.

A patient information leaflet about self-TWOC was provided to all the patients. In addition to that, they were instructed by the post-operative ward nursing staff on the procedure to ensure they could grasp the entire process before leaving the hospital. They were provided with a video recording link to assist them during the catheter removal process. Every patient was provided with a pair of disposable gloves, a syringe to deflate the catheter balloon, and swab packs to facilitate the catheter removal.

Catheter removal and troubleshooting

The TWOC date was determined by the operating surgeon after the operation. On the day of planned catheter removal, patients received a few questions on their smartphone application. The questions included queries about their general well-being, urine colour, ongoing urine collection in the bag, bowel movement, and the presence of any symptoms of fever or shaking/shivering. If they were safe, the application provided them with instructions to proceed with their catheter removal. Otherwise, it notified the virtual ward. During working hours, this means the elective post-operative ward gets informed. On receiving this notification, one of the team members communicated with the patient and provided necessary instructions. Additionally, the urology on-call team was contactable when needed. But out of working hours, all the communication was relayed through the urology team (on-call urology doctor). Patients were provided with contact details to communicate as required, in addition to the above-mentioned standard of practice.

Measuring environmental impact

Hospital catheter removal services are available at both of the hospitals under the Maidstone and Tunbridge Wells NHS trust. Additionally, community TWOC service is available in this NHS trust, which involves one of the service team members travelling from either site (e.g., MGH or Tunbridge Wells Hospital (TWH)) to the patient's home for the catheter removal. Hence, the environmental and travel-associated costs were calculated by the patient’s residence distance from the nearest of the two hospitals using Google Maps. Additionally, as a regional Holmium Laser Enucleation of Prostate (HoLEP) centre, we serve patients outside of the trust service area. For this group, TWOC is usually planned at Maidstone General Hospital (MGH), the primary urology service site. As a result, travel distances to MGH were calculated for these patients.

For the calculation of the carbon emission, the data published by the Department for Energy Security and Net Zero of the UK government has been used to collect the per-mile emission from an average car for different fuel types [10]. Additionally, data has been collected from the data published in early 2025 by the Driver and Vehicle Licensing Agency (DVLA) to calculate the distribution of different fuel-driven car percentages licensed in the UK [11]. With these, we have tried to best represent the environmental impact of the service in the discussion.

## Results

In this study, retrospective data were collected from 180 patients between July 2024 and June 2025. Due to the procedures predominantly being male surgeries, only 10 patients (5.6%) were female. They had TURBT (n = 9) and cystodistention (n = 1) procedures. The initial 44 patients comprised the pilot cohort, who were contacted to provide feedback. As this cohort widely accepted the service, feedback data were not collected afterwards.

**Figure 1:**
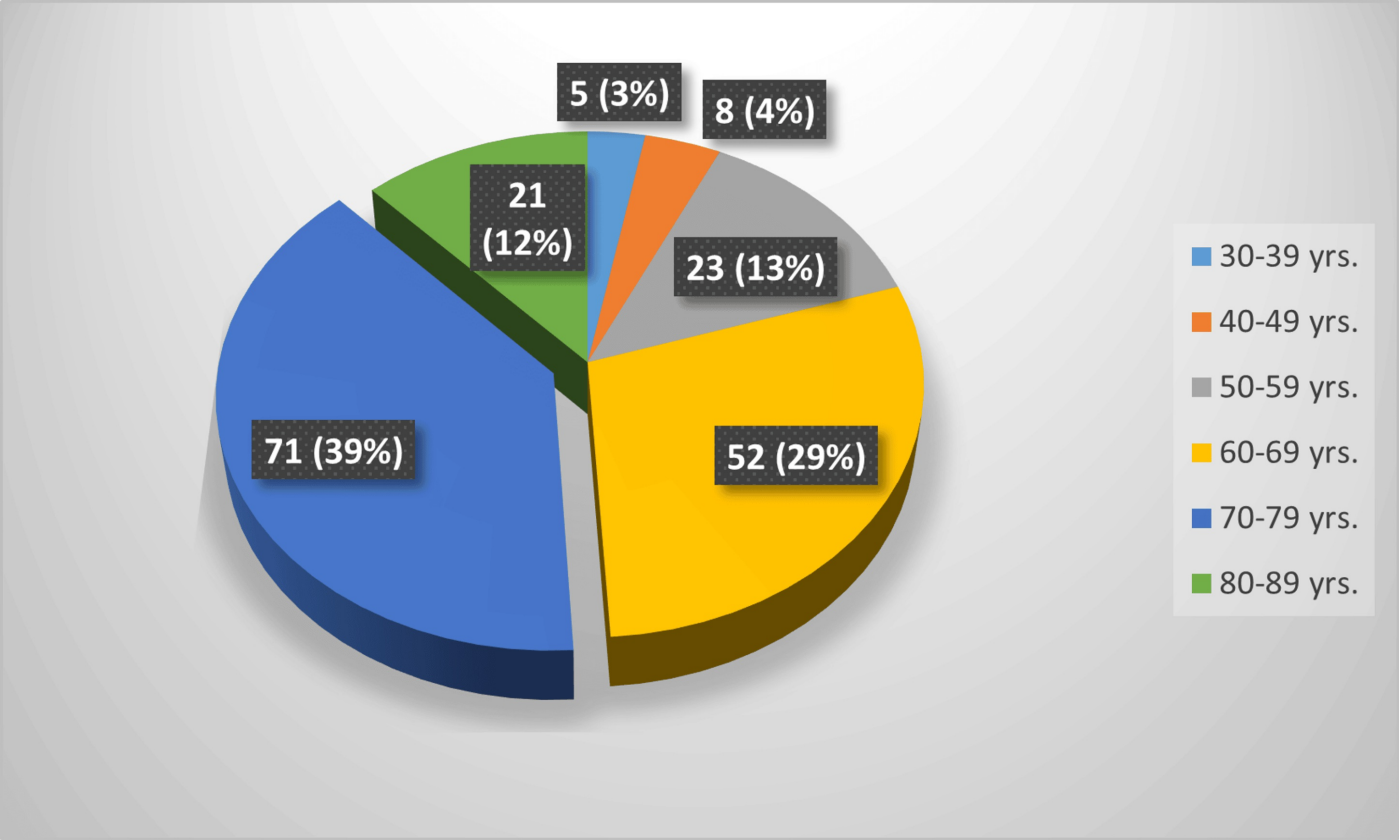
Patient distribution in different age ranges Colour-coded age ranges are shown in the right box. Total patient number: 180; labels represented as number of patients (percentage)

In this study, a five-point Likert scale was used for the quality of the mobile application and leaflet. The same scoring was obtained for the catheter removal experience of each patient. A question was asked about how likely the patient would recommend the service to others and whether they would undergo another self-TWOC in the future if needed. Apart from these scores, free-text feedbacks were collected. The patients taking this survey were in the age range of 36 to 85. Among them, 40% of participants were in the 70- to 80-year-old age range. Followingly, about 30% were in their 60s, 14% were over 80, and 12% were in their 50s. The distribution of patients across the age range for the total cohort is presented in Figure 1; the percentage is identical in the pilot cohort.

Approximately 74% and 59% of the respondents rated the leaflet and application service as excellent. However, there has not been a score of less than 3 for the leaflet received, but four patients found that the application does need better quality. On the other hand, the catheter removal experience was very smooth for 67% of the respondents, and there have been no reports of significant difficulty (Table 1).

**Table 1:** Patient feedback scores on the quality of the leaflets, virtual ward health platform application, and their experience with self-removal of the catheter removal. Feedback received from pilot cohort of 44 patients

	Five-point Likert scale	N (patient numbers)	Average score
Leaflet	5-Excellent	31	4.6
	4-Good	7	
	3-Fair	4	
	Have not rated	2	
Application	5-Excellent	24	4.2
	4-Good	7	
	3-Fair	6	
	2-Need improvement	4	
	Have not rated	3	
Catheter removal	5-Very comfortable	28	4.5
	4-Comfortable	8	
	3-Fairly manageable	6	
	Have not rated	2	

**Figure 2:**
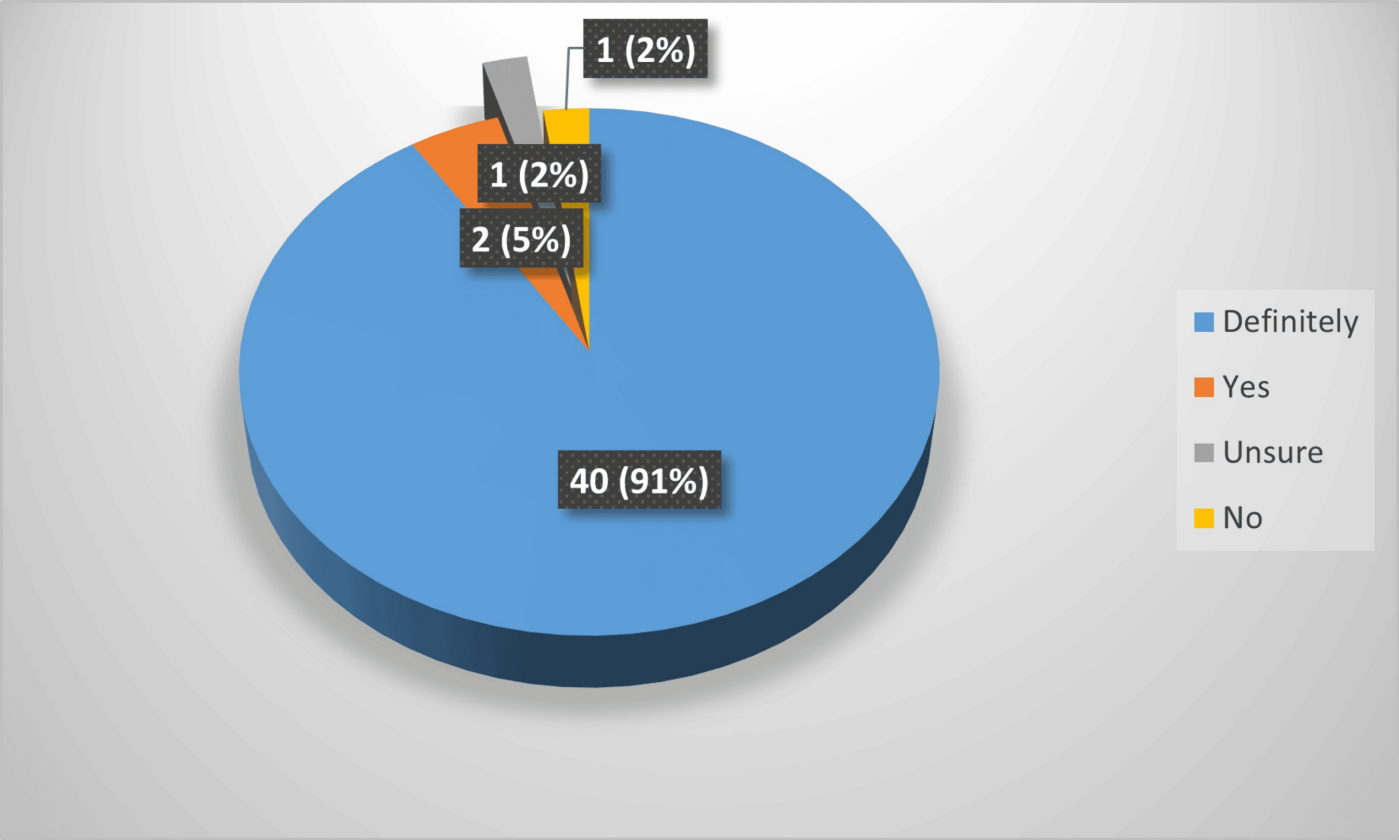
Pie chart showing patient feedback on the question 'How likely are you going to refer others to have the service?’ Colour-coded answer options are shown in the right box. Feedback was collected from the pilot cohort of 44 patients; labels represented as number of patients (percentage)

While responding, how likely they were to recommend the service to others, 96% of the patients were happy to recommend it to others. Among them, 91% will highly recommend others. Due to this overwhelming positive response, self-TWOC was adopted as a regular service after four months. These statistics are represented in Figure 2.

However, an ongoing registry has been maintained to track the events resulting from either the virtual ward being triggered by the application or the patient contacting the hotline to resolve their queries. This covers immediate (<30-day) problems after the self-TWOC. That has been evaluated, and a summary is presented here.

The most encountered problem was having haematuria (> grade one). As a result, TWOC was deferred for seven patients, and all of them had successful subsequent TWOC. For catheter-associated discomfort and bladder spasms, four patients had early catheter removal as they fulfilled the other criterion. Apart from these, one patient was contacted as they were experiencing sweating in response to a known side effect of regular medication. One patient had constipation and was treated accordingly, and then they had a successful removal. Seven patients had issues related to the application; among them, five were related to technical issues, which had been resolved. But two patients had difficulty using the application, hence supported by the team, and had a successful TWOC event. One patient in the cohort lost some of the provided equipment and was able to successfully remove the catheter with the advice over the phone. Two patients contacted due to post-procedure mild tenderness and were given appropriate advice to resolve the symptoms.

A total of 180 patients who underwent self-TWOC at home were successfully able to remove their catheter at home. The success of the procedure was determined by the patient's ability to remove their catheter at home and manage the natural passage of urination without requiring travel to the hospital or attendance by a community TWOC team member.

**Figure 3:**
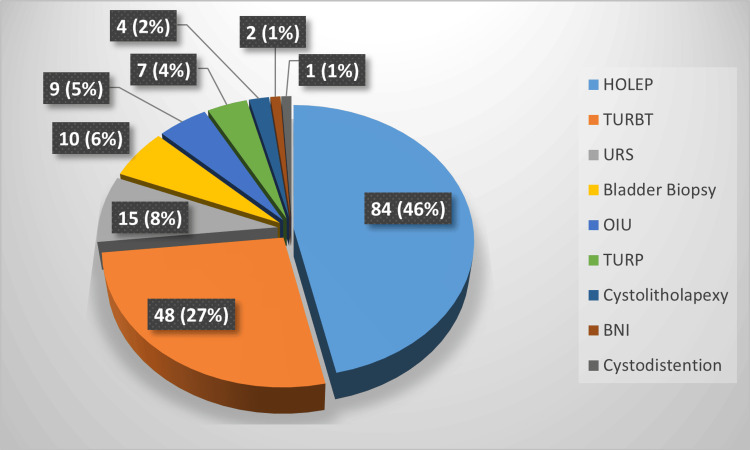
Pie chart representing the percentage of procedures for the included patient cohort Colour-coded procedures are shown in the right box; total patient number: 180; labels represented as number of patients (percentage)

As represented in Figure 3, about half of the procedures for the included patients were performed to treat bladder outflow obstructions. As a tertiary centre of HoLEP, 46% of the total cases were enucleation of the prostate with Holmium Laser. Another 4% were transurethral resection of prostate (TURP), and about 1% were bladder neck incision (BNI). Another principal share of these patients had bladder tumour procedures like TURBT, which consists of 27% in our study, and bladder biopsies in 6% of the patients. Apart from these, the patient had undergone urethral stricture procedures, including optical urethrotomy, urethral dilatation, cystolitholapexy, and cystodistention.

About 5221 miles of travel were saved by this initiative, about 29.2 miles per patient. This is roughly about 3 litres of average fuel saved for everyone [12], which may vary according to the type of fuel used, which amounts to about 14.02£ worth of travel cost saving for an average car in the UK [13]. Along with that, patients coming to a hospital TWOC need about 85£ spent by the NHS for the clinic slot [8]. Furthermore, each patient saved about 6.3£ for parking while attending the hospital [14]. These appointments are essential for the patients who were out of the area for HoLEP. Apart from the travel cost of the community TWOC team from two sites in Maidstone and Tunbridge Wells NHS Trust, community nursing team members who carry out and monitor the patients at home are needed. They are reported to have about 15£ per hour of average wage [15]. Whereas no additional cost was incurred for this self-TWOC service, as the virtual ward and existing monitoring technology were already in place for the Trust. Again, the personnel needed to contact patients in this project were managed using the existing staff of the post-operative short-stay ward, and we did not need an additional dedicated member for this service.

To achieve the closest possible calculation of carbon footprint offset to reality, we have collected data on the distribution of different fuel type cars in the UK. According to the DVLA data from early this year, 57% and 35% of the total fleet in the UK are petrol and diesel driven, respectively. Whereas, the share of hybrid electric, plug-in hybrid, and battery electric vehicles was nearly 3%, 1%, and 3.7%, respectively. The remaining 0.3% of the fleet consisted of liquid petroleum gas (LPG), compressed natural gas (CNG), and other types of fuel. The UK government data showed that the carbon emissions of different fuel groups vary from 0.34 emissions of carbon dioxide per mile for petrol or diesel to 0.26 and 0.22 for hybrid and plug-in vehicles. The electric vehicles had emissions of 0.09 per mile due to the average energy mix in the UK electricity grid. For calculating the weighted emission, the formula used was {(carbon emission per mile X total mileage saved) X percentage of fleet}⁄100. After adding them as detailed in Table 2, the total emission saved was 1.7 tons. This is higher than the per capita emission of more than 36% countries in the world. Furthermore, it is equivalent to the carbon emission offset from a London to Dubai flight for four passengers travelling in economy class [16].

**Table 2:** Percentage of the UK fleet of ‘average cars’ with respect to their type of fuel, with calculation of weighted emission for each type of vehicle with sum of carbon emission for 180 patients’ catheter removal-related travel kg CO2e: total greenhouse gas emissions expressed as equivalent kilograms of CO₂; EV: electric vehicle; others (~av): average for other small groups of fuel types

Type of fuel	Percentage of UK fleet	Carbon emission per mile (kg CO2e)	Weighted emission (kg CO2e)
Petrol	57	0.34	1011.83
Diesel	35	0.34	621.299
Hybrid	3	0.26	40.7238
Plug-in	1	0.22	11.4862
EV	3.7	0.09	17.38593
Others (~av)	0.3	0.36	5.63868
Total carbon footprint offset (in kg CO2e)			1708.363

While calculating the impact of self-TWOC in the HoLEP patients, we discovered that, compared to a year of data on the length of stay for these patients before the enrolment of self-TWOC, 91 patients among 220 were discharged on the same day; in comparison, eight months of data after the initiation of this programme enabled 115 patients among 210 HoLEP patients to be discharged the same day. As a result, our day-case rate for HoLEP rates increased from 41.4% to 54.8% with self-TWOC. This amounts to about 24 patients being discharged the same day of the operation, above the past trend of same-day case rate. It has been reported that the overnight stay of a patient might cost on average £400 [17]. Hence, in these eight months, about 9,600£ was saved by this project by increasing same-day discharges.

## Discussion

NHS England is experiencing a rising trend of urology day cases. From January 2017 to June 2022, there was a significant increase in day-case rates for BOO surgeries, rising from 8.3% to 21.0%. In 2021/2022, 92 out of 117 Trusts saw improved day-case rates compared to 2017 [2]. As a result, there is an acute rise in the necessity of TWOC services. Hence, it is a very important study that suggests the feasibility, safety, sustainability, and efficacy of self-TWOC for this group of patients.

There have been studies in the past on female pelvic surgeries in the United States of America [7,18-20], where self-TWOC is non-inferior to the hospital removal service. There has been an analysis in the NHS for urogynaecology post-operative patients as well, where 97% of the patients were successful in removing the catheter at home [17]. In urological procedures, which involve surgical intervention in or access through the urethra, there has been only one publication showing a 100% success rate following RARP, but these patients need to stay in the hospital following the procedure [8]. In this study, we have been able to demonstrate for the first time that self-TWOC is a safe process to remove the catheter in patients undergoing urology day cases, where we had 180 patients, who were able to remove their catheter without a hospital visit or a visit from a healthcare professional to their home.

Patient experience was very favourable. We found 96% of the patients preferred to refer others to have a self-TWOC service. This is comparable to the study of self-catheter removal in other groups of published sources. There were 97% patients in the RARP and 98% in the urogynaecology surgery group of patients expressing their wish to have the procedure again, if needed in the future [8,17]. Hence, it suggests a similar range of high acceptance in this study, apart from the comparable age distribution in other studies [17]. The results of this study suggest the safety of the group of patients over 70, who constituted the majority of the participants. The free-text response from the patient was overwhelmingly positive, and that is well represented by the results section. 

There has been a reported carbon emission offset of 0.01-0.02 tons per patient in England reported by Abou Chedid et. al; our calculated average of 0.01 tons/patient is comparable [8]. But it might vary with the demographics of the locality and the area covered by the centres across the world. Again, we have made our calculation in line with the national average of cars and their fuel distribution to the updated national data, which has not been done in any previous publications, which might differ in actuality, and can differ with time and change in composition of sources for energy generation in a country. Again, we did not spend any additional funds for this service, but application in a wider cohort might incur additional costs.

One of the concerns of self-TWOC is that early discharge of the patient might lead to adverse outcomes. Hence, in our service, we have used our virtual ward service, which offers personalized care by integrating close support with technology, enabling hospital-level care at home, as highlighted in NHS England's January 2023 'Delivery plan for recovering urgent and emergency care services' [21]. With this initiative, we have managed to free up outpatient spaces for the use of other patients, and effective resource management was achieved by reducing significant time of in-person clinician support with the use of artificial intelligence augmented virtual ward support. Although the patient helps with virtual clinical assessment and advice is still mostly clinician-driven in this project. But the help of technology did act as a safety net for a significant number of non-clinician-supervised patients. But access to the clinician team support was crucial to achieve safe service.

Furthermore, the selection of patients was largely dependent on the surgeon’s preference and experience with available previous data on voiding functions, which might be one of the key reasons for the success. Hence, further study to ascertain measurable and generalizable criteria might be helpful to replicate this model of service provision elsewhere safely and effectively, with the potential to spread this service to other patients needing catheter removal service. Moreover, this was a single-centre experience, without a comparator arm of conventional supervised TWOC, limiting generalisability. Follow-up focused on the immediate peri-operative period; longer-term outcomes were not assessed, which might be complicated by multiple factors and the development of other pathologies or aging. Finally, exclusion criteria are formed to ensure patient safety. And to further include those who could not use this application by themselves, options were extended to receive help from any of the patients' carers. In the future, studies that include patients who lack access to smartphones might be helpful to avoid this selection bias.

## Conclusions

This study demonstrates that self-TWOC, supported by a virtual ward, is safe, feasible, and well-accepted by patients undergoing day-case urology procedures when patients are properly selected. In our cohort of 180 patients, all successfully removed their catheters at home without complications, highlighting the potential reliability of this approach. However, further analysis is needed to identify the objective criteria for safely selecting patients for this service. By achieving this, self-TWOC could become a highly useful and scalable option for catheter removal care. It has the potential to increase day-surgery rates, reduce inpatient admissions, contribute to sustainability by lowering carbon footprints, and empower patients with remote monitoring, allowing them to recover in a familiar home environment while avoiding the hassle of acquiring catheter removal services.

## Appendices

Patient feedback questionnaire

Self-Catheter Removal Service

Thank you for choosing Maidstone and Tunbridge Wells NHS Trust for your self-catheter removal. Your feedback is invaluable in helping us improve our service. Please answer the following questions based on your recent experience. Your responses will remain confidential.

Information Leaflet

Please rate the information leaflet you received before your procedure.

How would you rate the quality and clarity of the leaflet?

·       Excellent

·       Good

·       Fair

·       Poor

·       Very poor

If you have any comments or suggestions regarding the leaflet, please share them below:

Mobile Application

Please rate your experience with the mobile application for virtual ward/self-catheter removal.

How would you describe the ease of use for the service?

·       Excellent

·       Good

·       Fair

·       Needs improvement

·       Poor

If you have any comments or suggestions regarding the application process, please share them below:

Catheter Removal Experience

Please rate your comfort during the catheter removal procedure.

How comfortable did you feel during the removal?

·       Very comfortable

·       Comfortable

·       Fairly manageable

·       Uncomfortable

·       Very uncomfortable

If you wish to elaborate on your experience or have any suggestions, please share them below:

General Feedback

If you have any other comments or suggestions to help us improve our self-catheter removal service, please write them here:
